# Circling Knowledge

**Published:** 1857-08

**Authors:** 


### CIRCLING KNOWLEDGE,

As to pecuniary condition, the movement is after the manner of the see-saw, now we go up, up, up, then we go down, down, down; but fashion delights in the circle, with most unequal radii, as erratic as those of the most incalculable comet. But knowledge is also circular. The present age knows a thing, the next succeeding forgets it; anon, a new generation springs up and claims it as a new discovery. We do not wonder that knowledge and invention and art, often died out in the night of ages, when there was no printing press to stamp it on the recorded page. But that useful knowledge should now dissolve almost from our view, and then be resuscitated with the claim made and the palm yielded for a new discovery, is at least of curious interest.

Within a year, the papers have announced it as the discovery of a Frenchman, that if a scion of a tree, or slip, has one end stuck into a potatoe, it seldom fails to grow. One of our earliest remembrances is of making the experiment with willow twigs. And even at that early day, we were familiar with the reason, that the moisture was better retained; we presume a turnip or parsnip, or beet or carrot, would answer quite as well as a potatoe.

Within a few days, another new discovery is announced as having come from France, for which high laudation is given to the ingenuity and perspicacity of M. Lundestorm, for making matches which rats cannot make go off, which cannot "go off" themselves, nor poison children, nor fill our premises with villainous smells. The wood is dipped in chlorate of potash, which, when applied to a surface covered with a preparation of phosphorus, (which having been kept at a high temperature for several days, becomes red and looses all its poisonous qualities) instantly ignites. A quarter of a century ago, before the modern match was ever heard of, we daily witnessed this striking of fire in the chemical lecture-room, by Prof. R. PETER, and now it is gravely announced, that "A paper was submitted to the Society for the Promotion of Industry, at Paris, which pointed out the evils and remedy of phosphorus matches, the practicability of which has been tested by several scientific men." O yes, certainly!

Within a few years, hatching chickens by artificial heat, was paraded as a French discovery. The Chinese have practiced it for centuries.

There is much of this new discovery in medicine, that is no discovery at all, and instead of being new, is as old as the hills. The cures of various forms of fever, the breaking up of fever and ague, by the application of cold water, is heralded by the disciples of Preisnitz, as a new thing, as a mark of the progress of the age. All these things were told us in our youth, as the experiments of Allopathic practitioners of ages before. But considerations of humanity allowed the practice to fall into desuetude. Cold water is a too powerful, and a too dangerous remedy for ordinary use, and safer, surer, and more available means have been sought for. The ignoramus, the charlatan, the quack, the imposter—these are the people who trifle with human life. The educated practitioner acts with a steady and cautious deliberation; with him, the life of a fellow creature confidingly placed in his keeping, is too holy a thing to be placed at the risk of random experiment or hap-hazard conjecture.

The regular practitioner, feeling the incompetence of his art in many instances, makes common cause with his brother, and considers it the business of his life to hunt out new and still safer, and more efficient remedies, and as soon as discovered, he throws his knowledge into the common fund, and the whole world is the wiser. But then there comes along, now and then, the mere semblance of a man, he, too, makes common cause as long as he is on the receiver's side, but the moment he discovers a gem, he secretes the treasure, and under the darkness of the night, carries it away to his own mean home, gloats over it awhile, then proclaims to the world its real, but oftener its only pretended virtues, its miraculous powers of cure. But what it is, he keeps to himself. He practically proclaims, "Come to me, or die." To place it in the hands of those who were once his brethren, that each one might aid the suffering in his own community, and thus its saving benefits be felt by myriads, who otherwise must perish, he refuses. And, with remorseless repetition, he exclaims still, "Come to me, or die." For this reason is it, that the regular physician looks upon those who keep their remedies to themselves with unutterable loathing, and if their names are ever uttered, it is but to express a depth of scorn and contempt which ordinary phrases fail to reach. And yet there is a meaner crew still farther down; festering beneath all these, in their own appropriate rottenness—the men who claim for a remedy the virtue which they know it does not possess. And of a brotherhood are they, who profess, but do not truly give the constituents of their remedies, in order to have themselves ranked among regular practitioners, giving their fabrications some popular designation, when, in some cases, there is not an atom of the article named, in a gallon of the preparation; coming within these categories, are the two "cathartics and pills," whose proprietors' names meet our eyes in almost every paper we take up, secular or religious, daily or weekly, headed by some historical incident, or some startling expression altogether inconsonant with the main subject of the article. "INHALATION" includes *all* these.

